# Child health services research with the people who know best: experiences and lessons from CanChild’s collaborations with families

**DOI:** 10.3389/fpubh.2026.1761542

**Published:** 2026-04-10

**Authors:** Elizabeth Chambers, Andrea Cross, Danijela Grahovac, Debra Hughes, Samantha Micsinszki, Monika Novak-Pavlic, Kinga Pozniak, Peter Rosenbaum, Rachel Teplicky, Vanessa Tomas, Marilyn Wright

**Affiliations:** 1 CanChild Centre for Childhood-Onset Disability Research, Faculty of Health Sciences, McMaster University, Hamilton, ON, Canada; 2 Department of Paediatrics, McMaster University, Hamilton, ON, Canada

**Keywords:** childhood disability research, childhood disability, family engagement, lessons from engagement with people with lived experience, partnerships with families

## Abstract

In the twenty-first century, granting organizations and journals increasingly expect health services research to include the active participation and voices of people with lived experience in studies relevant to their lives. This paper reports the 30-year history of engagement between CanChild Centre for Childhood-Onset Disability Research and families of children with neurodisabilities. We offer a brief background history of how these activities began and report on several programmes of research, knowledge sharing, and implementation that have benefitted enormously from the relationships with families and the insights they have provided on virtually every dimension of our work. We also briefly report on some of our recent formal and informal engagements with young people with lived experience, an aspect of our work that is now being more actively developed. We hope that this overview of one centre’s experiences and lessons learned from active partnerships with “the people who know” will be of value to others.

## Part I: Setting the stage—background and context for this article

“You have textbooks, we have story books!” (1)

Founded in 1989, CanChild Centre for Childhood-Onset Disability Research has always placed families at the heart of its mission. From the very beginning, our work in childhood disability—rooted in our expertise as clinically trained health services researchers—has been shaped by the voices and perspectives of families. In fact, the name *CanChild* was inspired by a parent, and later, the phrase “I am a CanChild!” was coined by a child partner.

This essay reflects our core philosophy of meaningful engagement with families and young people with lived and living experiences from the earliest stages of identifying clinical and health service priorities. We believe that families’ and young people’s insights are essential—not only in defining the issues that matter most, but also in framing research questions, designing studies, measuring impact, and translating knowledge into practice and policy. Our commitment is clear: families and young people with lived experiences are partners in every step of the journey.

When referring to “families” throughout this study, we refer to adults in caregiving roles who are responsible for the ongoing daily welfare of children. These are usually “parents” with a direct biological connection to the children, or adoptive, step, or foster parents, but may include other people in the child’s orbit, e.g., grandparents, aunts, and uncles with continuing responsibility for the wellbeing of the children. Throughout this essay, we refer to youth/young people—self-advocates with lived and living experience between childhood and adulthood. Foundational to the programme illustrations described in this study is the co-design with families. Co-design is a philosophy, with accompanying principles and practices, that embraces co-led and co-produced programme and research designs with interested parties, such as families, youth, researchers, clinicians, etc. (2, 3). It involves building relationships; centring the experiences and needs of those the programmes are intended to impact; power-sharing; and co-creating meaningful outputs through a variety of collaborative strategies (4).

In this review, we are pleased to provide stories about the co-development of several of the programmes we offer and to bring together and share some of the lessons we have learned. We hope that this report will be useful to others.

Regarding the ethical considerations related to this report, this article is an invited perspective and does not report new studies with human participants. Any references to surveys, focus groups, interviews, or evaluation findings related to previously published work are cited accordingly; ethical approval and participant consent were secured and are reported in the original publications.

## Part II: Examples of CanChild programmes co-created with families

### How we started—exploring family centred service (FCS)

Family centred service (FCS) is a philosophy and an approach to working with families that has informed all our work with families since CanChild’s inception. Very early in CanChild’s development, colleagues from Ontario’s Children’s Treatment Centres (CTCs), a consortium of community-based, publicly funded regional ambulatory service programmes, approached us for help to better understand these ideas. They had heard about “family-centred care” (FCC) and wanted to know what this meant and looked like. Despite already being involved in related work, we were unable to provide a clear answer. Thus, as one typically did in that era, we started by reviewing the literature to see what academic experts said about FCC. We were able to identify a long list of concepts and behaviors that were reported to characterize FCC. Still, we could not identify a coherent overview to help us distil these ideas into a clear answer for our colleagues. Nor was it evident that the academic experts had actively engaged with caregivers in the work they were reporting.

Having aggregated over two dozen apparently unconnected “elements” of FCC from our initial exploration, we had a novel idea: we turned to the family leaders of two provincial Cerebral Palsy family support groups to canvas their members. The result was a structured survey, completed by over 70% of their 280 members, whose perspectives on both the *perceived importance* of these individual literature-based FCC concepts and the subsequent *ranking of their relative priorities* provided invaluable insights for us.

The next step was to meet separately in focus groups with three CTC-based parent groups. Building on insights from the survey, we asked parents to describe what these key elements of family-centred care (FCC) looked like in practice—specifically, how each element felt when it was implemented well in their own experiences. After compiling a list of almost 400 ideas, we were able to refine the list and identify common themes and exemplars of these concepts. This enabled our research team to publish a conceptual framework describing Family-Centred Service (FCS) (5) and to create, test, and validate a measure of parents’ experience of FCS (*Measure of Processes of Care, or MPOC*) (6).

These activities proved to be the early steps in what quickly became an ongoing three-decade-long programme of research into a variety of issues concerning FCS. Our research programme explored FCS to assess whether and how it mattered to families (6–8); to know how best to engage in knowledge translation (KT) and sharing; to create a self-report measure for service providers to reflect on their family-centredness (*MPOC-SP*) (9); and to create teaching materials in an effort to respond to international invitations to present our work and support implementation and assessment of FCS. Our parent self-report measure of FCS (*MPOC*) (6) has been translated into >20 languages and is widely used for service evaluation both in Canada and internationally (10). For example, in Canada, it has been used by children’s rehabilitation centres and provincial disability programmes to evaluate the services they provide to families.

We have continued to work with parents to keep current on what families need and how these needs can best be met by services as the healthcare context changes. An integrated team of researchers and four parent co-investigators recently engaged nearly 70 parents in focus groups and interviews to learn about their experiences with healthcare delivery. Based on their insights, we revised our measure of family-centredness and developed a suite of knowledge translation materials on FCS for service providers and organizations. These materials—co-developed by family co-investigators and researchers, based on input from community families—constitute a collection of best-practice service ideas about what families need, and what practical actions service providers and organizations can take to make these things happen (11–13). We have also teamed up with a local organization serving racialized refugee newcomers to Canada in order to ensure the cultural safety and relevance of these resources.

We hope to see all children’s programmes and services offered in a family-centred, relevant way. In the following sections, we describe several other CanChild programmes, all co-designed with families and young people and rooted in the philosophy of FCS. We report how they were developed through authentic partnerships between families, young people and researchers, based on parent-identified needs, and how these programmes have had meaningful impacts on practice and policy.

### CanChild’s F-words programme

The *“F-words for Child Development”* (also known as *“My Favourite Words”*) constitute one of CanChild’s most successful programmes of research and knowledge translation. The programme began with a whimsical paper by Rosenbaum and Gorter (14) to bring the World Health Organization’s ICF framework for health (15) to life. Six “F-words” (family, fun, friends, functioning, fitness, and future) were mapped onto the ICF components and purposefully selected to be meaningful to children and families. The F-words capture everyday concepts that are easy for both families and service providers to understand across services and sectors. The definitions of the words, which have evolved over the years based on feedback from and conversations with families (e.g., redefining “Friends” and “Future”), brought forward ways to make the concepts more clinically relevant and useful for children and youth with disabilities and their families and service providers (16).

What grew out of the original article has become a full-scale, integrated, worldwide implementation programme (17). The CanChild F-words team now partners with families, youth, service providers from healthcare, education, social services, and community organizations, community members, and policymakers around the world. We have seen how the implementation of the concepts can expand and transform service delivery beyond the traditional medical model (focused on fixing) to promote a holistic, strengths-based, and function-focused approach that emphasizes living life and “doing stuff” (18–21).

The question that many people ask is: how did a simple study evolve into a worldwide phenomenon? The obvious answer is the efforts of our family partners/leaders and the commitment of our team to work together, to privilege and learn from the experiences of families and young people, and to consistently reflect on ways to improve our ideas and make these concepts into real-life applications. Building on the groundswell of interest in the F-words’ ideas, an integrated research team was formed in 2014 to focus on ways to disseminate these concepts. Essential to this team were several family champions who had learned about the F-words and were eager to share them with others. For example, the integrated team developed a 3-min video to share the F-words’ ideas more broadly. The video had over 700 views in two months and was positively reviewed (17, 22). This success led us to develop a workshop for organizations that was co-delivered by family members, healthcare providers, and researchers to increase knowledge and practical application of the F-words (17). The integrated team also developed practical, useful tools for families and young people, as well as an F-Words Knowledge Hub. With >100,000 unique visitors in 2025 alone, it has become the go-to resource for all things F-words.

The co-designed approach is the foundation of CanChild’s F-words Programme, which is expanding nationally and internationally. Our latest initiative and current CAN-Implement F-words Programme (23)—an F-words Implementation Support Programme (including training, educational materials, implementation coaches, and a community of practice)—is another example of this connectedness, which involves family co-leads and is being tested in Canada with plans to move internationally in the coming year. Families co-facilitate F-words Foundations and Implementation training, support F-words implementation planning, and offer invaluable insights and guidance to organizations—all to enable F-words use in practice. Additionally, expanding on educational offerings, with families and service providers globally, we recently co-developed an online self-paced F-words Foundations Training Programme, which has >3,400 graduates (18).

Family partners have led the way and guided the F-Words programme’s evolution and ongoing development. These family partners focus on bringing the F-words directly to other families, healthcare providers, educators, and community leaders, using real-life stories that display impact and elicit raw emotion. It is impossible to ignore them! Their enthusiasm for this approach and their genuine voices of experience have helped families to see how these concepts may reshape their lives through a strengths-based, holistic lens—offering empowerment and confidence. Family leadership and advocacy for an F-words approach have also shown providers the impact of what they do for/with children and families, beyond their technical skills. We have learned and shared that focusing on real-life activities that are meaningful to the child and family can open doors to setting goals based on things that people want to do, not just activities within a therapy context.

The long-term success of the F-words has been seen not only in practical application by families, healthcare providers, and educators, but also in policy. In Canada, provincial ministries have included the F-words as key pillars of policy documents related to the delivery of children’s development and rehabilitation services (24). These policy documents have been influential in promoting the adoption of these ideas across the sector, and we work actively to continue to build relationships with policymakers in ways that will advance adoption and effective use of the F-words and related family-centred, strengths-based ideas.

Having family partners co-lead CanChild’s F-words Programme has made a significant difference in the lives of other families and has influenced service delivery globally. The real-life examples that families provide are compelling and essential reminders of the realities they experience. The F-words programme would not be where it is today without invaluable guidance and leadership from families. As families have shared, “It’s a good way to remember how to advocate for your children and easier for others to understand your advocacy in a structured framework”, and “It’s been huge for us, although they are just six simple words; they’ve just changed the whole way we look at living” (18).

### ENVISAGE programme

CanChild-based service providers and researchers have long-standing interests in the wellbeing of families raising children with developmental concerns and disabilities (25–30). We, and many colleagues, have long been aware of and concerned about the mental and physical health impacts experienced by caregivers whose children’s lives were complicated by neurodevelopmental conditions. These disparate conditions threaten parents’ wellbeing and impose high costs in terms of time, energy, and worry. The findings of these many studies led us to want to explore how to address these known impacts on families parenting children with developmental conditions and particularly to wonder whether some of this well-described “morbidity” might be prevented. With colleagues and parents at the Australian Catholic University (ACU), where one of us (PR) was a Professorial Fellow from 2014 to 2017, we began to create a major research programme.

The project, directed to primary caregivers, was called *ENabling VISions and Growing Expectations (ENVISAGE)*. From the outset, ENVISAGE was co-developed with parents as equal partners, co-led and co-facilitated by parents, and then studied, analysed, and co-reported with parent partners. The original program comprised five-hour-long virtual workshops for small groups of parents of children under the age of 6 with any neurodisability, supported by learning materials and activities on our learning platform. In focused online synchronous discussions, a parent and a paediatric clinician share contemporary ideas about health and development; parenting as a dance led by the child; looking after oneself with self-compassion; and ways to connect, collaborate, and communicate these concepts with others in the families’ orbits (31).

During the original creation of ENVISAGE, our team of parents, clinicians, and researchers sought input from “the experts”—families of children with disabilities. Their lived experience was essential to ensuring that we captured key features that would help us structure the programme content. We used an online focus group in each country to discuss parents’ insights, priorities, and experiences. We provided additional opportunities for asynchronous feedback using a private Facebook group and a parent support organization. Not only did the parents’ contributions provide the basis for the programme’s concepts, but these interactions also served as networking opportunities to start building our team with several parents keen to partner on this endeavour (31, 32). These parents and clinician-researchers worked together over the course of several months to develop the initial programme content and consider its delivery format. To assess the feasibility of the initial iteration of the programme (its clarity, acceptability, and practical usefulness), our team sought feedback about the content and structure of the programme from additional parents not involved in its development on the programme’s content and structure. After incorporating their recommended changes, the pilot programme was launched, co-facilitated by a parent–clinician team in each country, and delivered nationally. The findings of the Families’ programme were powerful and impactful (33). Parents reported an increased sense of empowerment and greater confidence in their parenting roles. At the same time, there were other benefits and opportunities that we could not have anticipated at the outset. These led to several further developments of the ENVISAGE programme.

In addition to their constructive input about the original Families programme, many parents strongly recommended that the ENVISAGE team develop a service provider analogue (which has since been created and validated as *ENVISAGE-SP*)! Parents in the Families programme had rightly argued that while it was valuable to have the knowledge, tools, language, and lenses that ENVISAGE had offered them, it was essential that the service providers with whom they worked had the same set of concepts and resources so that together they could speak the same language and work collaboratively on the family’s goals. This took the “dance” metaphor (about parenting) to a whole new level with service providers dancing with and being led by families in their care. The development of the ENVISAGE-SP programme followed the same developmental phases as parents, clinicians, and researchers adapted the Families materials to address a healthcare provider audience. Again, the programme was co-delivered by a parent–clinician team. Although service providers recognized the concepts as familiar, the programme’s strength was in packaging these ideas into a coherent framework supported by an evidence base. Service providers appreciated the programme’s family-centred, strengths-based, holistic emphases. Moreover, the discussion format, with parent co-facilitators, allowed service providers to process these ideas more deeply and consider how to apply them in their own work context. The findings from this study are currently being prepared for publication.

The reach of these programmes has differed across countries, depending on specific national opportunities. For example, Australia’s Department of Social Services has awarded the ENVISAGE-Families programme ASD 11.9 million to roll out across the country over five years. In Canada, based on service providers’ experience in the pilot programme, there is now active engagement with some children’s health and development centres in Ontario and Alberta who are keen to train their staff in ENVISAGE-SP and offer the ENV-Families programme to their client families.

Another spinoff was the international creation and adaptation of the ENVISAGE programme. Together with families, a then-doctoral student at CanChild (MNP) created a Croatian version of the ENVISAGE-Families programme (34). What began as a PhD thesis project has now evolved into a programme of research over the last decade, with multiple community outreaches and continuous contributions from a group of Croatian family champions. From the early beginnings, Croatian parents of children with developmental disabilities, along with researchers and clinicians, worked side-by-side to adapt the programme’s content and delivery. Their lived experience and insights enabled us not only to translate ENVISAGE but also to establish it as a culturally sensitive, appropriate and meaningful parent empowerment programme in the Croatian community. As this ongoing collaboration continues to grow into implementation projects that now reach hundreds of Croatian parents, we are reminded that when nurturing a culture of shared learning, co-creation, and systemic change, we are opening up new opportunities that extend far beyond individual families and the direct benefits they might see; we are also mobilizing communities and creating a world in which we value families and what matters to them. As more international teams take on the same ENVISAGE endeavours in their respective countries, *ENVISAGE-Croatia* serves as yet another powerful example of the global impact of a bold idea 30 years ago at CanChild—to have families at the table—on the landscape of childhood disability research worldwide.

### Family engagement in research (FER) programme

In 2011, patient and family engagement in research was recognized as important in health research across Canada. For example, Canada’s major funding body, the Canadian Institutes of Health Research, launched its Strategy for Patient-Oriented Research. This document defines patient and family engagement as “working with patients and families as equal partners and valued members of research teams” (35). It was acknowledged that collaboration was clearly needed, but at the time, there were few effective strategies for how to do it. Because researchers and families come from very different worlds, bridging this gap can be challenging. Both sides may feel nervous—worried about being misunderstood, feeling unsure about differences in knowledge and power, and dealing with the awkwardness of early conversations as families and researchers figure out how to communicate and work together.

In conversations with other researchers and family partners in the field of neurodevelopmental disability, it became apparent that, despite a shared belief in the value of meaningful engagement, we did not know how to do it well. There was a need for training for both researchers and families. In 2017, our CanChild team received funding from the Kids Brain Health Network (a national organization) to create a training programme to fill this gap. After eight months of course development led by families, trainees, and researchers, the Family Engagement in Research (FER) Course was launched (36). With this 10-week online Certificate of Completion course offered through CanChild, McMaster University supports families, researchers, and healthcare providers to partner together in research and foster authentic connections that centre lived and living experience alongside academic expertise. Through our innovative co-learning and community-building model, families and researchers co-learn in small community-specific cohorts, building strong relationships and acquiring new knowledge and skill sets.

The first six cohorts of the FER course (2018–2021) underwent a pre- and post-evaluation to assess learners’ perceptions and satisfaction with the course and its impact on their perceived knowledge, abilities, attitudes, and self-confidence in FER. The findings showed statistically significant increases in graduates’ perceived knowledge, abilities, and self-confidence in engaging in research. Attitudes towards family engagement in research were consistently rated high at baseline and post-course completion (37). Follow-up with FER course graduates two years post-course found sustained knowledge and skill outcomes, and many had increased involvement in FER since the course (38).

At the time, our goal was to train 40 people—20 researchers and 20 family members. By the end of 2025, we had over 700 graduates from 23 countries. What began as a bold idea to bring families and researchers together in the same online “classroom” has become a movement—one that is reshaping child health and neurodevelopmental research to be more inclusive, responsive, and impactful. The programme has grown to include other educational/training programmes, such as foundational workshops, a second Certificate of Completion (the Family Engagement Leadership Academy), and a growing community network (e.g., social media, a Community of Practice, and a newsletter). As the FER community grows, so too does interest in adapting and implementing the course in graduates’ local contexts. Through international collaboration, the FER course has been translated and delivered in Brazilian Portuguese and Dutch and scaled to Australia. A study funded by the Canadian Institutes of Health Research (CIHR) is currently underway to explore the adaptation of the FER course for youth with neurodevelopmental disabilities and chronic health conditions after a needs assessment was completed in 2023 to understand the training needs of youth (ages 18–25) with neurodevelopmental disabilities (39).

Through our training courses at McMaster University, our growing international network, and our ongoing support services, we are building capacity for meaningful engagement between families and service providers that extends far beyond the classroom. We believe that when families are authentically engaged in research and healthcare, the outcomes will be better for everyone.

### *About My Child* (a suite of family report tools)

The “About” suite of tools integrates the tenets of family-centred service, family empowerment and engagement, and relationship building through communication, collaboration, and connection. These parent-report questionnaires provide families the opportunity to share a fuller picture of their child and family from their unique perspective. *“About My Child”* enables parents and caregivers to reflect on and share their child’s and family’s strengths and interests through five F-word-based questions. Parents then (i) indicate whether they are “concerned about” any of 20 areas of child development and functioning, and if so, (ii) consider the impact of noted concerns on their child’s “participation in everyday activities”, and (iii) add narrative comments to clarify or elaborate on their responses.

This strength-based, holistic, and non-categorical family report focuses on what a child can do, what is going well, and parents’ hopes, while recognizing and identifying parent-identified “concerns”, challenges, needs, and priorities. Scoring can reveal the cumulative functional “concerns” and needs to describe the “health complexity” of children.

The original *About My Child* tool *w*as developed in 2008 within the World Health Organization’s ICF framework (15). Reliability and validity were subsequently established (40). In 2021, based on consultations with family and colleague collaborators, the introductory “strength-based” questions were added. In response to feedback and requests, *“About Me”,* a youth self-report version, was developed. The adaptations were based on input received from youth, parents, and clinicians. Clinicians using *About My Child* as an intake tool also identified the need for a version more attuned to the needs of families with infants and toddlers. Thus, *“About My Baby/Toddler”* was created collaboratively with families and clinicians.

*About My Child* was initially developed for use in large-scale population surveys. More recently, the tools have been used clinically to identify strengths, needs, and priorities to inform service options, with the intention of supporting and optimizing a child and family’s wellbeing while building positive relationships. For example, the *About My Child* tools are currently being used in Ontario, Canada, as a guide for initial exploratory conversations at publicly funded SmartStart Hubs and partner organizations to connect families with appropriate child development services (24). However, all tools can be used for ongoing clinical practice, programme development, and research.

CanChild’s *Access and Equity Guide* can also be used with the *About My Child* tools as a conversation guide to explore a family’s contextual personal and environmental factors. The guide outlines areas known to be facilitators or barriers to accessing services. These include logistics such as transportation, language, access to technology, and multiple service needs; social determinants such as housing or food security; and family issues such as concurrent healthcare needs of other family members or family dynamics. This information can inform clinical decision-making and potential supports. Topics can be explored as appropriate and revisited as needed. The *Access and Equity Guide* is available free of charge on the CanChild website.^1^

### Youth engagement—early experiences of CanChild’s newest venture, now under development

While caregiver perspectives are essential, they are not meant to represent, let alone replace, children’s autonomy and self-advocacy. In our work, we have intentionally integrated youths’ lived experiences into co-production activities in developmentally appropriate ways. In the COVID Time Capsule project, youth with diverse abilities participated as youth research partners. Accessibility and belonging were prioritized: meetings were structured to be engaging and supportive, and individualized accommodations were implemented to enable meaningful participation. For example, one youth partner who is non-verbal contributed through tailored communication strategies collaboratively developed with the team. Youth partners co-developed a data collection tool grounded in the F-words approach and participated in reviewing and interpreting emerging findings. In this way, they were engaged as knowledge brokers. Ongoing efforts continue to expand structured, developmentally responsive mechanisms to further embed children’s and youth’s perspectives across initiatives.

Youth engagement has also been extended to include knowledge dissemination. For example, at the 2025 IAACD/EACD conference in Heidelberg, one of our youth partners presented a poster on F-words across the life course, offering firsthand perspectives on how concepts resonate and evolve from a youth standpoint. His work reflects on lived experiences and offers critical reflections on how strength-based language shifts in meaning over time. The F-words team actively supported this participation by reviewing the abstract before submission (as we did with and for one another) and by providing financial support to enable conference attendance. This experience positioned a youth voice not only within research development but also within international scholarly dialogue, reinforcing the importance of youth-led contributions in shaping and communicating disability research.

## Part III: Lessons learned

### Overview

Over CanChild’s 35-year journey of research in childhood disability, we have learned invaluable lessons (41). Our field’s traditional approaches to health services research involved trained “experts” (researchers and experienced service providers). We identified the issues that compelled us and explored them from our perspectives, applying our conceptual frameworks and methodological tools as we understood them. The best of that kind of work often produced useful outputs and insights; however, in our blinkered approach, we failed to recognize the limitations of our work—namely, that we represented particular perspectives and experiences. Over the past few decades, with the increasing expectation that clinical services, research, and advocacy all need the active engagement of the people who “know”—namely, those with lived experience of the issues of importance to all of us—we now expect to seek, respect, and value the issues and perspectives of the people “about” whom we had previously done research.

#### Genuine partnerships with families and young people

From the outset of a project or programme, families’ voices and concerns (and now more formally, the voices of young people with lived experience) actively inform the research that is conducted. Developing projects and programmes based on needs identified by people with lived experience (i.e., “Do people really need and want this intervention?”) builds credibility in the processes and outcomes. This in no way invalidates the observations we make as experienced service providers. What we have learned, however, is how important it is to seek confirmation from families and young people about both whether the impressions we form in the clinic are valid and what underpins these realities that families express. In other words, we need their insights into whether and how to explore these issues through credible research.

#### Complementarity of perspectives

From the start, it is essential to build a strong core team of people with complementary perspectives, including having people with lived experience “baked” into the team and programme from the start. It is important to note that our CanChild family and young colleagues have always brought a trove of lived experiences. Indeed, some of our treasured colleagues approached CanChild researchers because they were already aware of their interest in collaborating with similar people. People with lived experience sometimes include formally trained academics. However, many people with whom we work, who hold leading roles in various projects, have acquired technical and research-focused skills after being welcomed as people whose issues and concerns relate to specific topics, programmes, policies, and projects of mutual interest. They also bring a wealth of life experiences from their personal careers in fields such as education, business, and the arts, thus enriching all aspects of our academic work. Their established connections with disability communities provide valuable networks for accessing niche skill sets and to aid in participant recruitment and extend knowledge translation. Engaged families and youth are champions who can further the work by capitalizing on opportunities in their worlds that researchers may not even be aware of.

We are intentionally increasing our efforts to engage diverse perspectives and experiences in all our work. This is essential, given the context of our country, which includes racialized, newcomer, and Indigenous families and communities. Working with them, listening to them, and learning from them has challenged us to think differently and has helped expand and enhance our work even further!

#### Enduring networks of connections built on trust and respect

Many of our initial collaborations have evolved into lasting relationships that allow us, as researchers, to stay connected to relevant issues in families’ lives [e.g., networks of parents such as the Parents Partnering in Research Facebook group (42)] and for us, as researchers, to know we have an ever-increasing network of trusted and trusting potential advisors and community-based leaders to whom we can turn. These connections help us extend our reach beyond researcher-only approaches and into the communities where children and families live, and where we hope the best of our validated research findings will be taken up and applied. As a result, we believe that our research is likely to be more relevant, nuanced, and tailored to real life and hence more meaningful to the target community (and we have early evidence of that from our ENVISAGE programmes).

This genuine (and generous) engagement empowers everyone involved in the activities. It trains community members in research processes and researchers in collaborative approaches across traditionally separate silos of interest and knowledge. This opens up future opportunities that may not be apparent at the outset (an excellent example is the creation of the ENVISAGE-SP programme, something the original researchers had not imagined, but to which parents cued us!).

Initially, our impetus to address the issues described in this paper was to co-develop programmes or tools to support families or those who provide services to them. Over time, it became apparent that the scope of interest exceeded any team’s resources to support. In an effort to establish sustainability and extend the reach, we became increasingly convinced of the necessity of building capacity in families and service providers to carry the work forward. Parents who had worked alongside us, and those with a keen interest in joining us, were supported to become project leads, contribute to activities that suited their interests and availability, and provide feedback to improve CanChild’s work and reach in meaningful ways. They led the development of programming and facilitation training to build parents’ and clinicians’ skills. Capacity building, however, was not the only goal. We realized that relationship building was fundamentally important to this endeavour. We developed a shared vision, mission, and ethos. We trusted each other’s insights. We became friends and colleagues. This was, at its core, team building. It is important to note that among the authors of this report, EC, DG and KP are parents of young people with neurodisabilities.

#### Impact on KT and credibility

As expected, active collaborations with people with lived experience facilitate knowledge translation (KT). The real-life examples that families provide are compelling—and recognizable to others, including the policymakers with whom we enjoy productive and trusting relationships (24). Families’ contributions help us move beyond statistical significance and to truly understand and think about the meaning of research findings in the lives of children and families. The stories behind the numbers can tell us so much more and inform policy directions and future research. Our family partners connect with key individuals in their own spheres of influence. They know which mechanisms of KT are preferred and how best to convey and explain the relevance of our research findings to families. For example, one of our family champions with a wide social network in the disability community emerged as a valued knowledge broker for our team. Hearing the research findings from a trusted parent in their own community carries weight with both other parents and policymakers—probably even more than words from the ivory tower! The community’s trust is essential and should be addressed alongside that of colleagues, SPs, and policymakers. Thus, focused KT activities can increase the credibility of the findings with colleagues, service programmes, and policymakers, who are powerfully influenced when they can recognize the value ascribed to the research findings by the people *with* whom (rather than *about* whom) we are doing our research or knowledge translation work.

## To conclude: a parent collaborator’s perspective

Over the past three decades, CanChild’s work has consistently demonstrated that meaningful family involvement transforms research, practice, and policy. When families are recognized as experts in their own right—bringing deep, experiential knowledge of disability, systems navigation, and daily life—the focus of inquiry and service shifts from “doing for” to “working with.” Empowerment emerges when families are not only invited to participate but are equipped, trusted, and supported to lead. Across CanChild programmes, such as Family-Centred Services, the F-words for Child Development, ENVISAGE, and the Family Engagement in Research (FER) initiatives, families have played central roles in co-designing studies, co-leading workshops, shaping measures, and mentoring peers. These collaborations have shown that empowerment is a reciprocal process: as families gain skills, confidence, and a sense of belonging, researchers and service providers gain insight, humility, and a richer understanding of what truly matters. Empowered family members become advocates, educators, and innovators—helping to bridge systems, influence practice, and foster communities of shared learning and mutual respect. Ultimately, authentic family and youth involvement ensures that programmes and policies reflect lived realities, advance equity, and promote wellbeing for children, families, and the professionals who support them.

## Data Availability

The original contributions presented in this paper are included in the article/supplementary material; further inquiries can be directed to the corresponding author.

## Footnotes

1
https://canchild.ca/resources/?srch=access+and+equity+guide
